# Intranasal delivery of mitochondria for treatment of Parkinson’s Disease model rats lesioned with 6-hydroxydopamine

**DOI:** 10.1038/s41598-021-90094-w

**Published:** 2021-05-19

**Authors:** Jui-Chih Chang, Yi-Chun Chao, Huei-Shin Chang, Yu-Ling Wu, Hui-Ju Chang, Yong-Shiou Lin, Wen-Ling Cheng, Ta-Tsung Lin, Chin-San Liu

**Affiliations:** 1 Vascular and Genomic Center, Changhua Christian Hospital, 135 Nanhsiao Street, Changhua, 50094 Taiwan; 2 Department of Neurology, Changhua Christian Hospital, 135 Nanhsiao Street, Changhua, 50094 Taiwan; 3 School of Chinese Medicine, Graduate Institute of Chinese Medicine, Graduate Institute of Integrated Medicine, College of Chinese Medicine, Research Center for Chinese Medicine and Acupuncture, China Medical University, Taichung, 40447 Taiwan

**Keywords:** Biological techniques, Cell biology, Neuroscience, Diseases, Molecular medicine, Neurology, Pathogenesis

## Abstract

The feasibility of delivering mitochondria intranasally so as to bypass the blood–brain barrier in treating Parkinson's disease (PD), was evaluated in unilaterally 6-OHDA-lesioned rats. Intranasal infusion of allogeneic mitochondria conjugated with Pep-1 (P-Mito) or unconjugated (Mito) was performed once a week on the ipsilateral sides of lesioned brains for three months. A significant improvement of rotational and locomotor behaviors in PD rats was observed in both mitochondrial groups, compared to sham or Pep-1-only groups. Dopaminergic (DA) neuron survival and recovery > 60% occurred in lesions of the substantia nigra (SN) and striatum in Mito and P-Mito rats. The treatment effect was stronger in the P-Mito group than the Mito group, but the difference was insignificant. This recovery was associated with restoration of mitochondrial function and attenuation of oxidative damage in lesioned SN. Notably, P-Mito suppressed plasma levels of inflammatory cytokines. Mitochondria penetrated the accessory olfactory bulb and doublecortin-positive neurons of the rostral migratory stream (RMS) on the ipsilateral sides of lesions and were expressed in striatal, but not SN DA neurons, of both cerebral hemispheres, evidently via commissural fibers. This study shows promise for intranasal delivery of mitochondria, confirming mitochondrial internalization and migration via RMS neurons in the olfactory bulb for PD therapy.

## Introduction

The intranasal route of drug administration offers a new approach to treating brain/central nervous system (CNS) disorders, including Parkinson's disease (PD), Alzheimer disease, Huntington's disease, epilepsy, etc.^1^. It accelerates drug development because therapeutic substances traditionally delivered via intravenous, intraperitoneal, and oral routes are hampered from crossing the blood–brain barrier (BBB) to reach targets in the CNS. Nasal spray, nose infusion, or nasal inhalation are possible routes for nasal administration of drugs. However, the efficiency of drug absorption must be considered, depending upon the target to be treated. For example, a portion of a therapeutic agent administered via a nasal spray or inhaler enters the pulmonary circulation, reducing delivery to the brain through olfactory or trigeminal nerves, a shortcut for nose-to-brain targeting^2^. Intranasal delivery has advantages and disadvantages^3^. In addition to the rapid onset of action due to delivery of drugs to the CNS within minutes, efficacy of neurotherapeutics is enhanced and side effects are decreased by direct delivery of drugs to the brain with reduced or minimal systemic delivery. In contrast, drug properties (excluding conventional nasal medications), volume of solution, and molecular masses of drugs are restricted, and absorption via the nasal mucosa and brain-targeting pathways of drugs also vary, depending upon individual pathology and drug chemistry.

Mitochondria are vital, nano-sized organelles, and mitochondrial replacement therapy has been employed to treat mitochondrial diseases in clinical trials^4^. Thus, based on a similar treatment concept, mitochondrial transplantation has been extensively developed to cure diverse diseases in recent years^5^. Parkinson’s disease (PD), a common neurodegenerative disease with no cure, is associated with mitochondrial dysfunction, damaging dopaminergic neurons in the substantia nigra (SN) of the midbrain^6^, but mitochondrial transplantation raises the prospect of new treatments for PD. In situ or intravenous injection of naked mitochondria isolated from healthy cells has successfully restored mitochondrial function, with subsequent improvement of mobility in neurotoxin-induced rat models of PD^7,8^, but the feasibility and efficacy of intranasal administration of mitochondria, a relatively simple, safe approach are unknown. However, stem cells delivered intranasally enter the CNS^9^ and have been used in treatment of PD^10,11^ and malignant brain tumours^12^.

Treatment efficacy of mitochondrial transplantation depends on several variables, including how many mitochondria can be taken up by cells in a finite period, because mitochondria not internalized by cells gradually lose activity^13,14^, and because different delivery routes have different intake efficiencies^13^. Mitochondrial transplantation via appropriate approaches may accelerate active, instead of passive uptake via interstitial pressure, osmosis, or endocytosis^13^. It is especially suitable for diseases involving chronic neuroinflammation or pathogenic disruption of cytoskeletal function, decreasing the probability of mitochondrial intake via actin-dependent endocytosis^15,16^. Pep-1, a member of the cell-penetrating peptide (CPP) family, increases importation of foreign mitochondria by damaged cells, restoring mitochondrial function and reducing oxidative stress without prior chemical modifications for treatment of mitochondrial diseases such as Myoclonic Epilepsy Associated with Ragged-Red Fibers (MERRF) and mitochondrial myopathy, encephalopathy, lactic acidosis, and stroke (MELAS)]^14,15,17,18^ in vitro, or PD in vivo^7^. Unlike our previous study, which employed local injections of mitochondria for treatment of PD-model rats lesioned with 6-hydroxydopamine, in the present study, we explored the feasibility of intranasal delivery of mitochondria for brain targeting and compared it to mitochondrial transplantation with or without Pep-1 pre-modification in same animal model. In support of direct nose-to-brain drug/cell delivery for more effective PD treatment^19,20^, we propose that mitochondria may be a useful intranasal delivery system for non-invasive clinical treatment.

## Results

### Improvement of rotational and locomotor behavior

**Figure 1:**
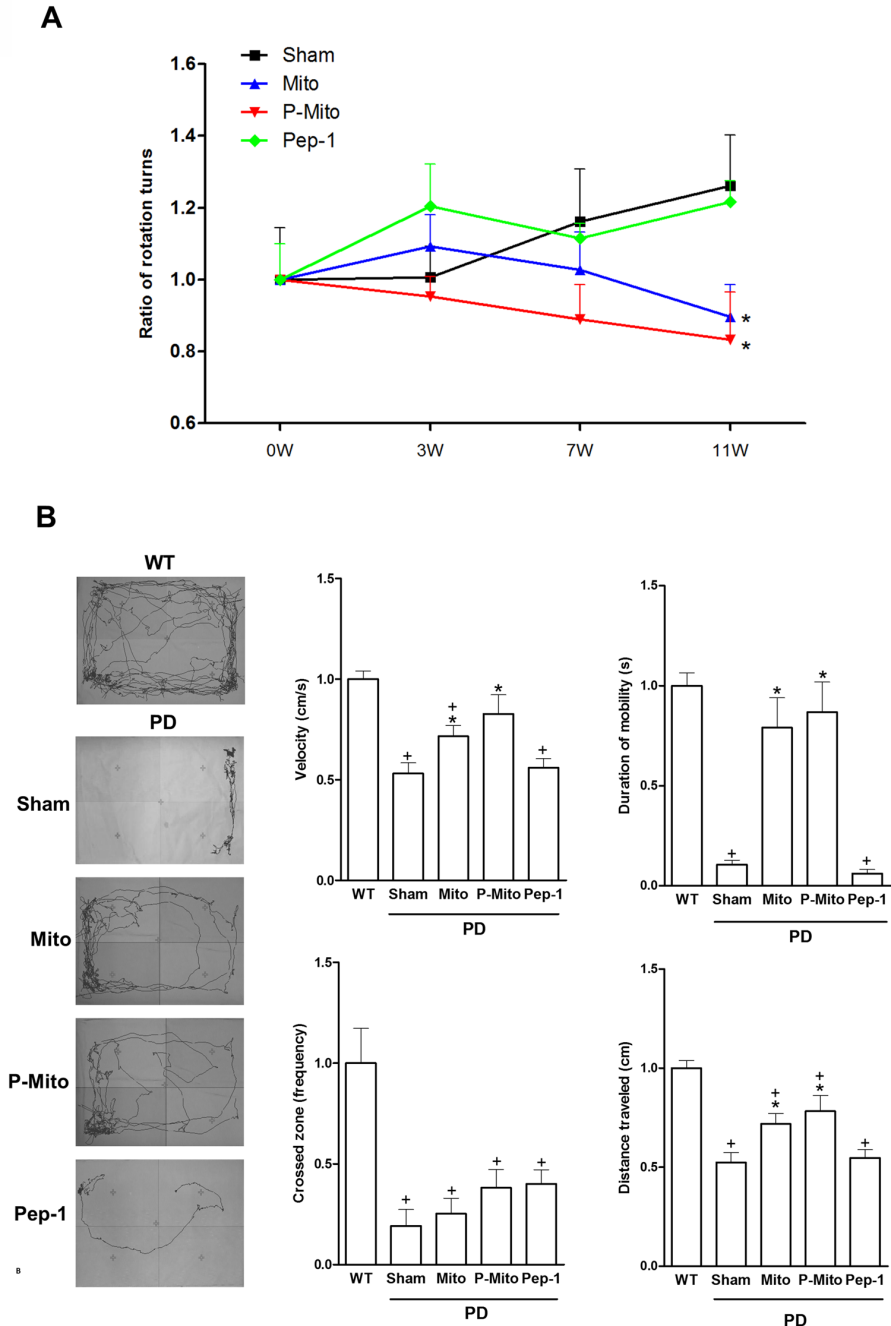
Three-month intranasal delivery of allogeneic mitochondria increases activity and improves behavior of Parkinson’s disease rats. (**A**) The rotational response to apormorphine in each experimental group relative to wild type was calculated by normalizing the number of ipsilateral rotations during a 60 min period. (**B**) Locomotor activity represents spontaneous movement in an open field during a 15-min observation period. The open-field test quantified the average velocity, zone-crossing frequency, duration of mobility, and distance traveled. In all graphs, values from treated animals were normalized to those of wild-type controls. +*P* < 0.05, vs. WT; *P < 0.05, vs. sham; *WT* wild-type controls, *PD* Parkinson’s disease, *Sham* vehicle alone, *Mito* mitochondrial alone, *P-Mito* Pep-1-labelled mitochondria.

Behavior of PD rats was assessed in terms of apomorphine-induced rotations (Fig. 1A) and performance in the open field test (Fig. 1B). Rotation data for the WT group are not presented due to the extremely low frequency of apomorphine-induced turning in normal rats (Fig. 1A). The Sham group showed a dramatic increase of apomorphine-induced rotational activity relative to the inactivity of the WT group (Fig. 1). Unilateral intranasal infusion (ipsilateral to the lesioned side) of Mito or P-Mito caused a significant decrease in rotational activity 11 weeks post-treatment (Fig. 1A) and recovery of normal locomotor activity at 12 weeks, as measured by mean velocity, mobility duration, frequency of crossed zones, and traveling distance, compared to the Sham group (Fig. 1B). There was no significant difference in behavior between the Sham and Pep-1-only groups, or between the Mito and P-Mito groups (Fig. 1).

### Support of nigral dopaminergic neurons against lesion-induced cell death in SN and ST

**Figure 2:**
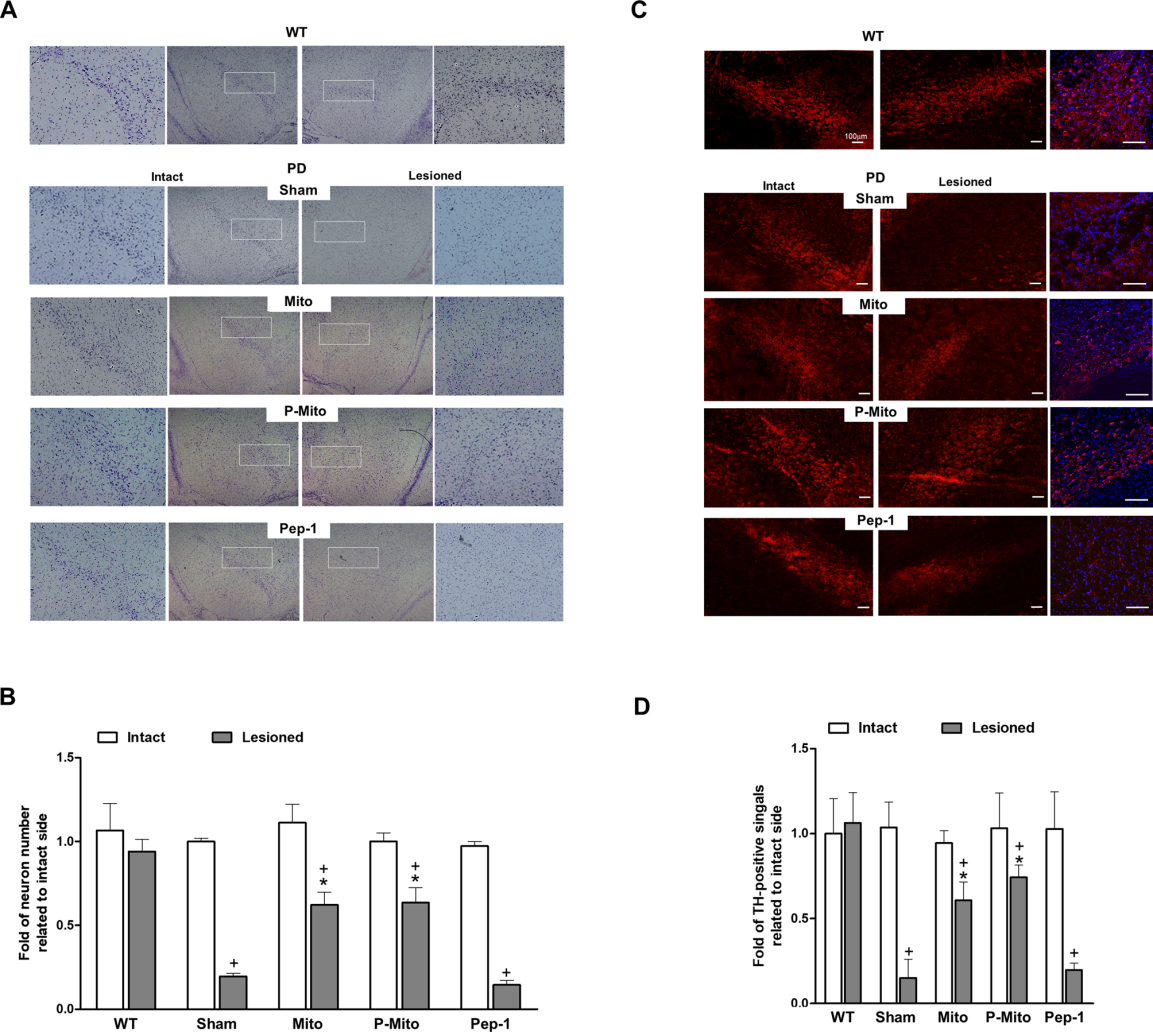
Mitochondrial infusions restore lost Nissl-stained neurons and dopaminergic (DA) neurons in the substantial nigra of Parkinson’s disease rats. (**A**) Nissl staining in coronal midbrain illustrates survival of neurons. The substantia nigra pars compacta (SNc) of the intact and 6-OHDA-lesioned sides, indicated by rectangles, was shown in high-magnification images in each left or right panel. (**B**) The number of Nissl-positive neurons was quantified by normalizing each contralateral control at the same magnification using ImageJ Software. (**C**) Slices were stained with tyrosine hydroxylase (TH) (red fluorescence) and 4′,6-diamidino-2-phenylindole (DAPI) (blue fluorescence) to identify dopaminergic neurons and nuclei in the SNc. High-magnification images of TH fluorescence with DAPI nuclear counterstaining in right halves of WT and PD brain are shown in third column. (**D**) Quantification of TH-positive signals was performed similarly to normalize each contralateral control at the same magnification. +*P* < 0.05, vs. Intact; *P < 0.05, vs. Sham; *WT* wild-type controls, *PD* Parkinson’s disease, *Sham* vehicle alone, *Mito* mitochondrial alone, *P-Mito* Pep-1-labelled mitochondria.

Neuron survival in the substantia nigra (SN) was examined by Nissl staining 3 months post-treatment (Fig. 2A, B) and survival of dopaminergic (DA) neurons was further confirmed by TH immunofluorescence staining (Fig. 2C,D). Magnified images of TH fluorescence with 4’,6-diamidino-2-phenylindole (DAPI) nuclear counterstaining in right halves of WT and PD brains are shown in the third column of Fig. 2C. Obvious asymmetry was consistently apparent in both Nissl-stained (Fig. 2A) and DA neurons (Fig. 2C) between the left (intact) and right (6-OHDA-lesioned) halves of the brain in the SN of the Sham group versus the WT group. In the sham group, there was a significant loss of both signals in the lesioned side of the SN, with an average survival of ~ 18.5% relative to the intact side (Fig. 2B,D). In contrast, a significant increase in survival of SN (Fig. 2B) and DA neurons (Fig. 2D) in the lesioned side of the SN was found in the Mito (µ = 68.4%) and P-Mito (µ = 73.2%) groups, with similar levels of performance indicating the effectiveness of mitochondrial treatment for neuron survival. No effect was seen in the Pep-1-only group (Fig. 2).

**Figure 3:**
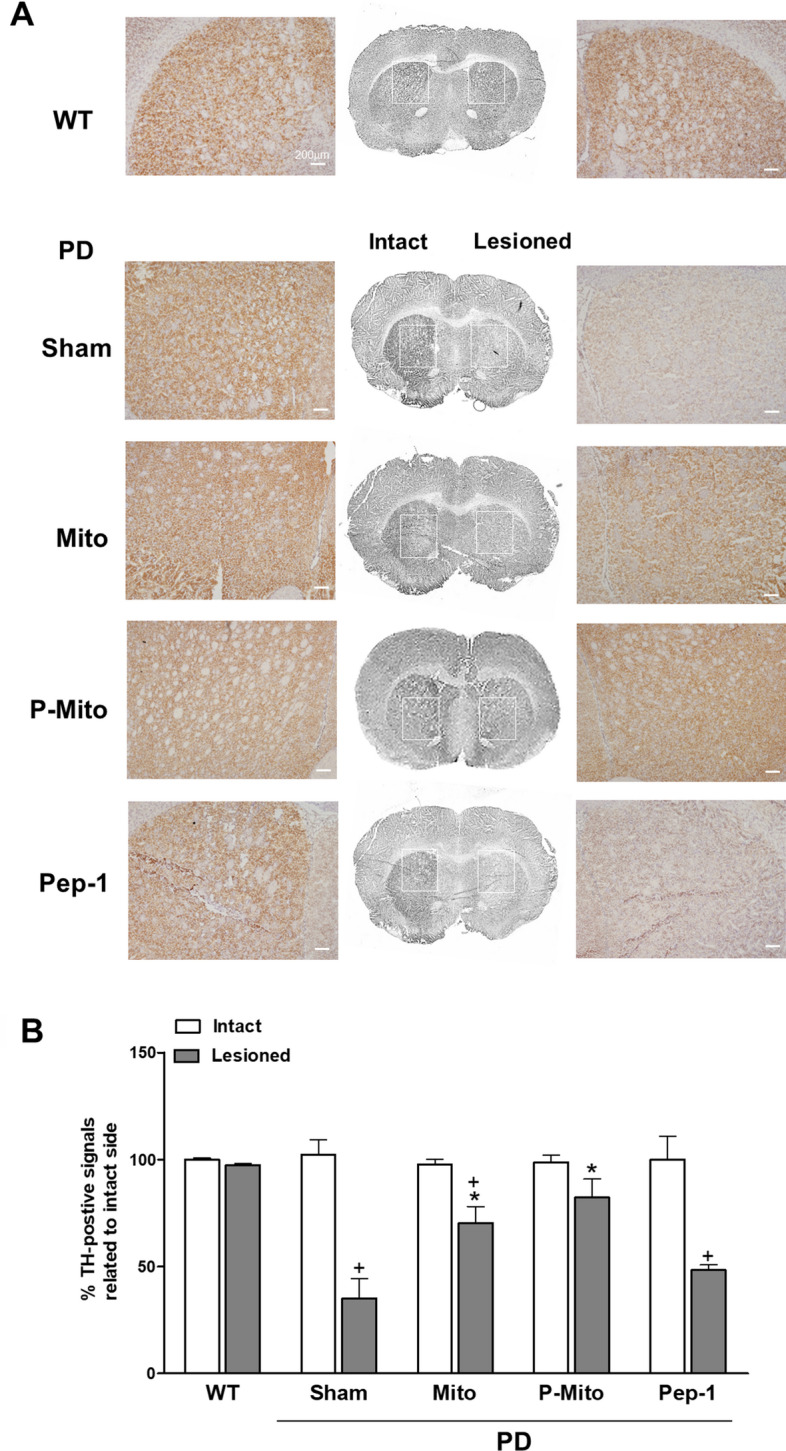
Mitochondrial infusions attenuate unilateral damage in nigrostriatal dopaminergic neurons of Parkison’s disease rats. (**A**) Immunohistologic staining of tyrosine hydroxylase (TH) was performed with coronal forebrain sections. TH expression (brown color) in striata (ST) of the intact and 6-OHDA-lesioned sides, indicated by rectangles, are further shown in high-magnification images in each left or right panel. (**B**) Levels of TH-positive signals in the striatum were evaluated with ImageJ Software and TH expression was quantified by normalizing each image against its contralateral control. +*P* < 0.05, vs. Intact; *P < 0.05, vs. Sham; *WT* wild-type controls, *PD* Parkinson’s disease, *Sham* vehicle alone, *Mito* mitochondrial alone, *P-Mito* Pep-1-labelled mitochondria.

Furthermore, immunohistochemical TH staining also showed consistent results in the striata (ST) of the Mito and P-Mito groups, which were comparable to those of the sham and Pep-1 groups (Fig. 3A). The significant loss of DA neurons that project to the ST, disrupting the motor circuit of the basal ganglia, was restored by Mito and P-Mito treatments, but not in the sham or Pep-1-only groups (Fig. 3B). The difference of TH performance between lesioned and intact sides diminished significantly in the P-Mito and Mito groups (Fig. 3B), though there was no significant difference between them (*p* = 0.08).

### Restoration of mitochondrial function in SN and modulation of plasma inflammatory cytokine responses

**Figure 4:**
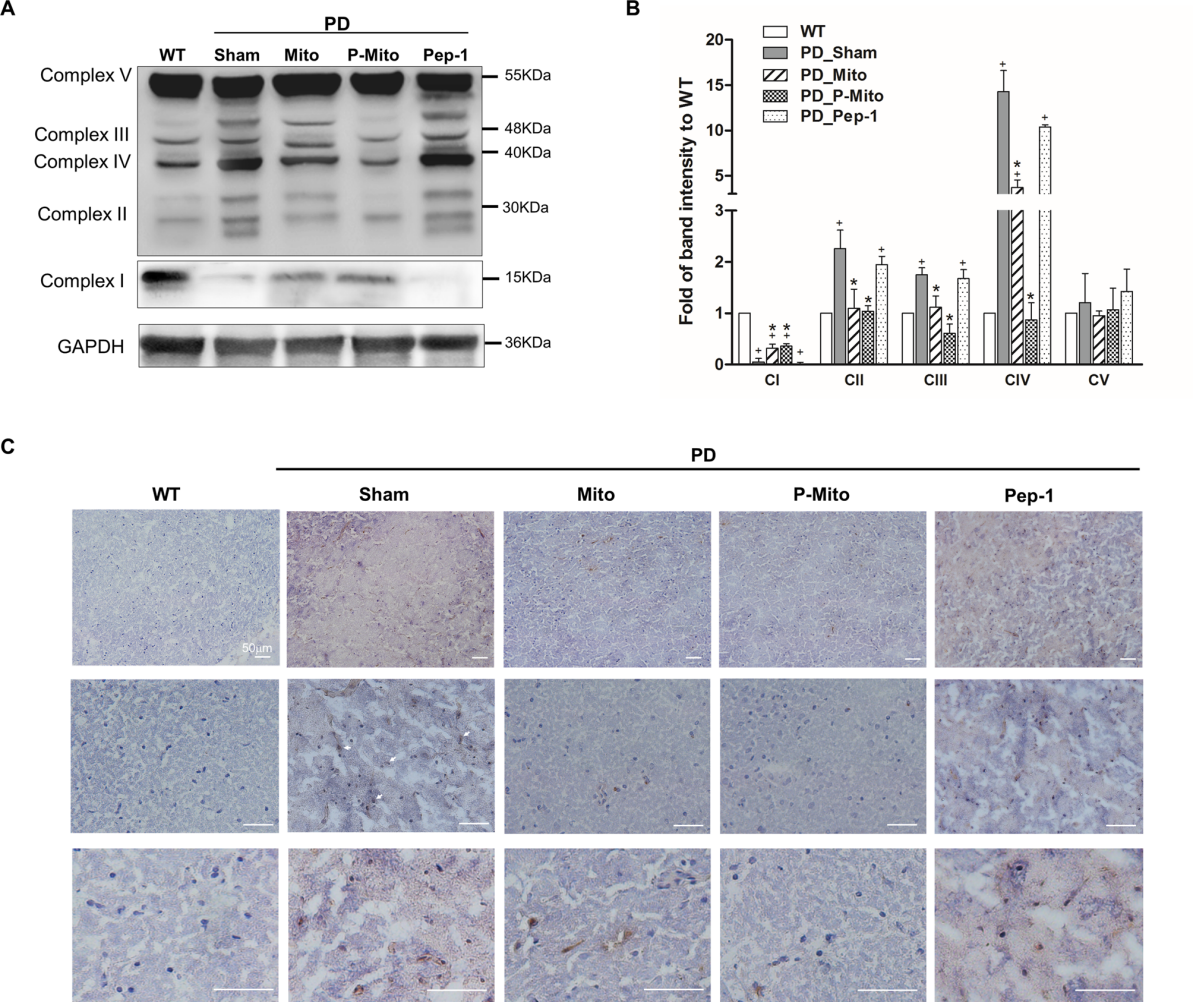
Normalized expression profiles of mitochondrial complexes in lesioned substantial nigra (SN) document subsequent attenuation of oxidative stress. (**A**) Mitochondrial integrity in the SN of the midbrain after 3 months of treatment was assessed and (**B**) quantified based upon protein levels of mitochondrial respiratory chain complexes I–V in same PAGE gel, normalized against an internal control, glyceraldehyde 3-phosphate dehydrogenase (GAPDH), with same amount of loading protein. Changes in the patterns of mitochondrial proteins, including band intensity and molecular weights, relative to wild-type controls, are presented. For whole uncropped images of original western blots with three independent samples of each group, please refer to Supplementary Figure (Fig. S1). (**C**) Immunohistological stain for 8-hydroxy-2'-deoxyguanosine (8-OHdG), a marker of DNA oxidative damage, was performed to examine DNA oxidative damage in the SN. Brown color indicates specific immunostaining of 8-OHdG (as depicted by arrows at high magnification, lower panels). Blue dots indicate nuclear haematoxylin staining. +*P* < 0.05, vs. WT; *P < 0.05, vs. Sham; *WT* wild-type controls, *PD* Parkinson’s disease, *Sham* vehicle alone, *Mito* mitochondrial alone, *P-Mito* Pep-1-labelled mitochondria.

Mitochondrial function in SN neurons was examined by analyzing expression of proteins of the electron transport chain and oxidative damage using 8-hydroxy-2-deoxyguanosin (8-OHdG) staining. Western blots showed an obvious loss of complex I (CI) in the SN of sham and Pep-1-only groups compared with the WT group. In contrast, complexes II–IV (CII-CIV) showed significant increases in band intensity and obvious shifts in molecular weight (Fig. 4A). Both the Mito and P-Mito groups showed patterns of mitochondrial complex proteins like those of WT, documenting a significant recovery of CI and explaining the performance normalization of CII–CIV relative to the sham group (Fig. 4A). Quantification revealed that CI-CIV substantially returned to normal in the Mito and P-Mito groups and that the adjustment in CIV expression in the P-Mito group was more significant than in the Mito group (Fig. 4B). Meanwhile, IHC staining of nuclei in SN neurons exhibited strong 8-OHdG signals (Fig. 4C) in the Sham group compared to the WT group, whereas nuclei were significantly less stained in both the Mito and P-Mito groups, though their staining levels were still higher than those observed in the WT group (Fig. 4C) .

**Figure 5:**
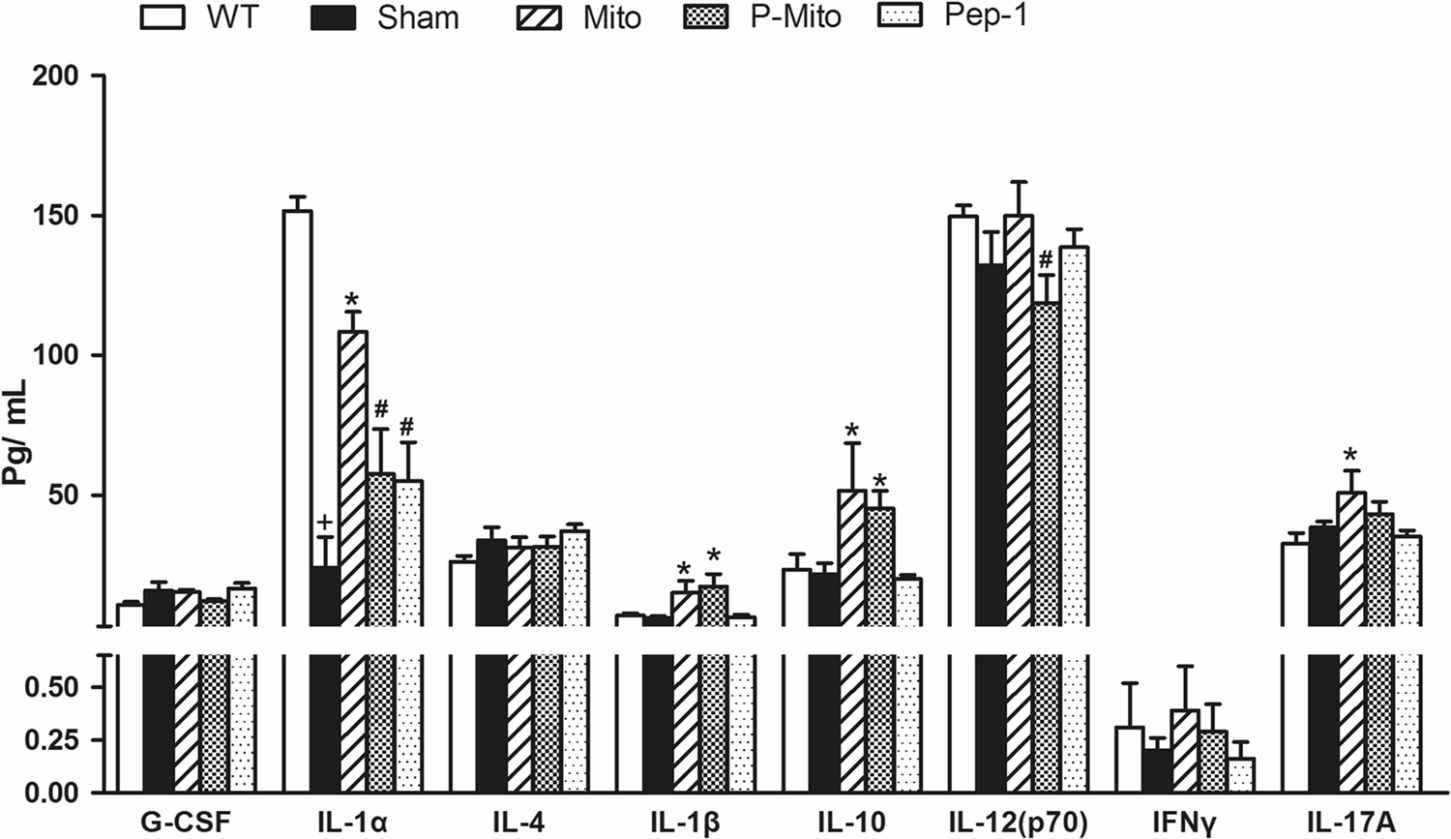
Pep-1 labeling of mitochondria reduces plasma levels of inflammatory cytokines. Plasma was separated from blood collected after 3 months of treatment. Cytokines of granulocyte colony-stimulating factor (G-CSF), interleukin (IL)-1a, IL-4, IL-1b, IL-10, IL-12 p70, interferon gamma (IFN-g) and IL-17A were selected due to involvement of grafted mitochondrial regulation in Parkinson’s disease rats, shown in our previous study^7^. +*P* < 0.05, vs. WT; *P < 0.05, vs. Sham; #*P* < 0.05, vs. Mito, *WT* wild-type controls, *Sham* vehicle alone, *Mito* mitochondrial alone, *P-Mito* Pep-1-labelled mitochondria.

There was no significant difference in plasma levels of inflammatory cytokines between WT and Sham groups, except for Interleukin (IL)-1alpha (IL-1α), which showed a significant decrease in the Sham group (Fig. 5). Compared to the Sham group, Mito treatment evoked a strong increase of inflammatory cytokines, especially IL-1α, IL-1beta (IL-1β), IL-10 and IL-17A. P-Mito treatment not only decreased expression of IL-1α and IL-17A as in the Mito group, but also suppressed IL-12 levels (Fig. 5). Thus, infusion of Pep-1-conjugated mitochondria further attenuated the plasma inflammatory cytokine response.

### Presence of allogeneic mitochondria in nigral dopaminergic neurons innervating the striatum through the rostral migratory stream

**Figure 6:**
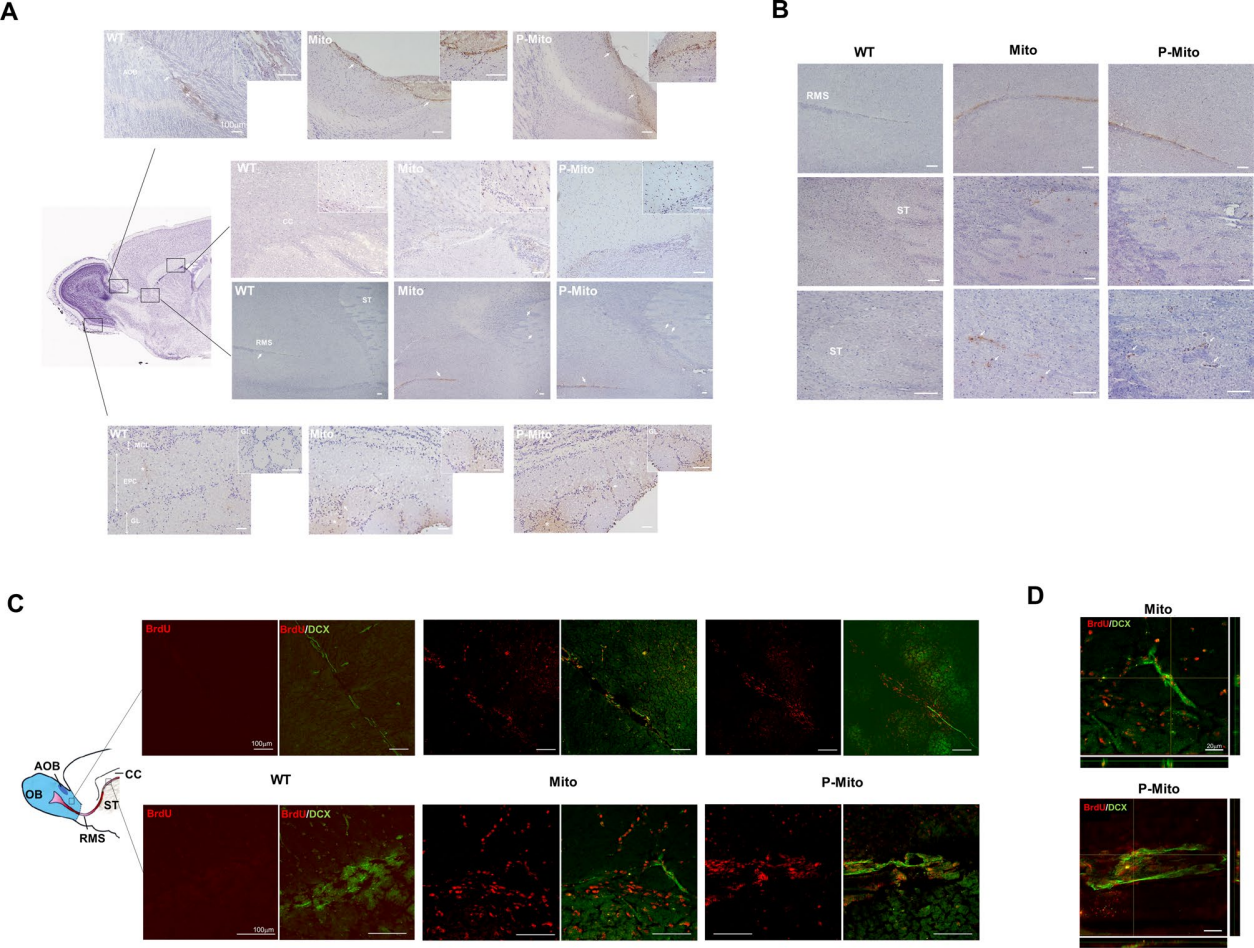
Representative photomicrographs showing distribution and delivery of intranasally administered mitochondria labeled with bromodeoxyuridine (BrdU) via the rostral migratory stream (RMS). (**A**) Anti-BrdU antibody followed with horseradish peroxidase allowed tracking of allogeneic mitochondria (brown color) in different brain regions of the olfactory bulb glomerular layer (GL), accessory olfactory lobe (AOB), corpus callosum (CC) and striatum (ST), represented as rectangular high-power fields in the sagittal sections of mouse brains. Nuclei were counterstained with Meyers's hematoxylin (blue dots). Arrows indicate BrdU-positive signals in high-magnification fields. BrdU-labelled mitochondria are obvious in the ST (third panel) and CC in zoomed images (second panel). Intranasally administered mitochondria penetrated to the AOB (first panel), but not to the bulb glomerular layer (GL) of the outer tissue layer of the olfactory bulb (fourth panel), and progressed into the RMS (third panel). (**B**) Expression of BrdU signals in the RMS and ST at higher magnification. (**C**) Double immuofluorescent staining with doublecortin (DCX) and BrdU antibodies confirmed the uptake of exogenous mitochondria (red color) in DCX-positive migrating neurons of the RMS (green color) around the AOB / ST-CC interface, indicated with a rectangle. (D) Co-localization of images was captured in double-labeled sections in single optical sections of z-stack confocal images, presented as side views of the x–z and y–z planes. *P < 0.05, vs. Sham, *WT* wild-type controls, *Mito* mitochondrial alone, *P-Mito* Pep-1-labelled mitochondria, *OB* olfactory lobe, *EPC* external plexiform cells, *MCL* mitral cells.

BrdU IHC images of brain sagittal sections (lesioned side) visualized the uptake of intranasally infused allogeneic mitochondria labelled with BrdU in different parts of the brain, including the corpus callosum (CC) and ST, accessory olfactory bulb (AOB), rostral migratory stream (RMS) track, and glomerular layer (GL) layer of the main olfactory bulb (Fig. 6A). Compared to the untreated WT group, despite having a mild antibody background around the AOB area (asterisks), mitochondrial treatment groups revealed the marked presence of allogeneic mitochondria in the region of an RMS-like track penetrating into the AOB. They also diffused into parts of the CC (Fig. 6A), as well as the ST (Fig. 6B). Expression of BrdU signals, excluding background noise, was relatively less visible in the GL layer olfactory bulb network and was hardly discernible in deeper layers of the external plexiform cells (EPC) and mitral cells (MCL), compared to the GL layer (Fig. 6A). For further confirmation of mitochondrial delivery via RMS neurons, double immunofluorescence labeling of migrating neuroblasts (doublecortin (DCX)- and BrdU-labeled mitochondria) showed exogenous mitochondria in DCX-positive neurons of the RMS in the area of the AOB / CC/ST interface (Fig. 6C) in the Mito and P-Mito groups, but not in the WT group. Colocalization of BrdU and DCX signals revealed by Z-stack sections confirmed internalization of allogeneic mitochondria in RMS neurons around the CC/ST area (Fig. 6D).

### Mitochondrial delivery to dopaminergic neurons of the contralateral striatum via interhemispheric commissures

**Figure 7:**
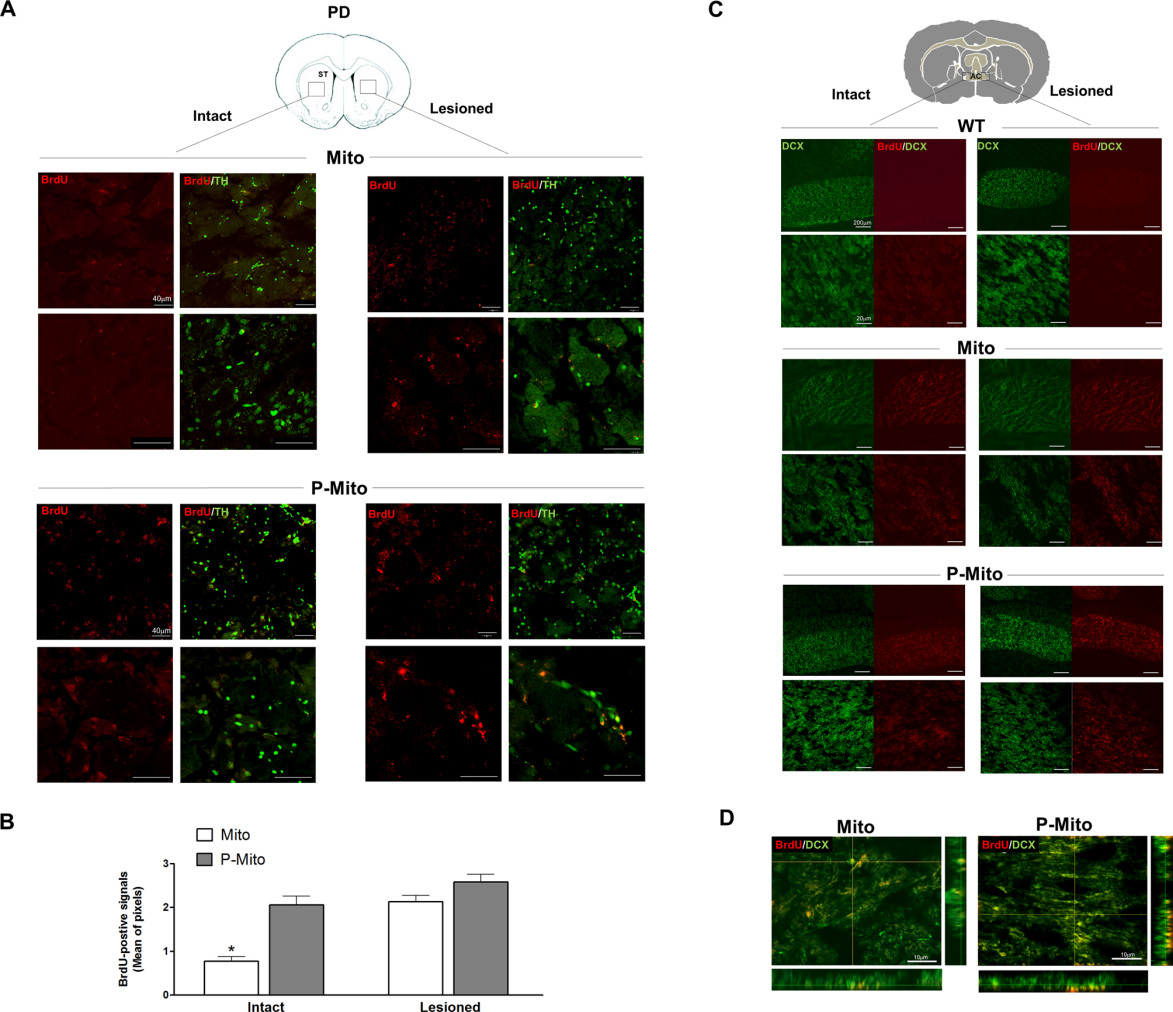
BrdU-labeled mitochondria were expressed in striatal DA neurons and DCX-positive neurons of the anterior commissure (AC), defined by cross-sectional areas of the forebrain. (**A**) Double immunofluorescent staining of BrdU (red) with TH (green) was performed to confirm intranasally administered mitochondria in striatal DA neurons and (**B**) BrdU signals were quantified individually in lesioned and contralateral (intact) sides using ImageJ Software. (**C**) Reconfirmation of contralateral delivery of mitochondria employed double immunofluorescent staining, using BrdU (red) with DCX antibody (green) in the intact and lesioned sides of the AC, a bundle of axons connecting the two cerebral hemispheres. Images were taken at different magnifications in each group. (**D**) The merged image of Z-Stack confocal images, shown as side views of the x–z and y–z planes, revealed the localization of exogenous mitochondria in DCX-positive neurons. *P < 0.05, vs. Lesioned side, *WT* wild-type controls, *Mito* mitochondrial alone, *P-Mito* Pep-1-labelled mitochondria.

Double staining of brain cross-sections with BrdU and TH confirmed the internalization of allogeneic mitochondria in nerve terminals of nigral DA neurons in the ST of Mito and P-Mito groups (Fig. 7A). Some TH-positive DA neurons possessed exogenous mitochondria that could only be seen at higher magnification (Fig. 7A). Notably, mitochondria from unilateral intranasal infusion were not only observed in ipsilateral ST of the lesioned side (Fig. 7A), but also in the contralateral side of the intact hemisphere (Fig. 7A). The latter were visibly more numerous in the P-Mito group than in the Mito group, as revealed by denser BrdU signals, but exhibited similar fluorescence in the lesioned side (Fig. 7A,B). To explain allogeneic mitochondrial internalization in both cerebral hemispheres, double immunofluorescence staining (DCX/BrdU) of migrating neuroblasts was performed in coronal brain sections of the decussation of the anterior commissure (AC) (Fig. 7C). BrdU immunoreactivity in the Mito and P-Mito groups revealed expression of foreign mitochondria in neurons of the AC between the ipsilateral and contralateral hemispheres. Mitochondrial transmission in AC neurons from the treated side to the contralateral (intact) side was also more obvious in the P-Mito group than in the Mito group (Fig. 7C), which was consistent with BrdU expression on the contralateral side in the ST (Fig. 7A). The Z-stack for 3D reconstruction at high-magnification merged confocal fluorescence images of DCX-positive cells on the lesioned side AC (Fig. 7D). Co-expression of DCX and BrdU signals in merged images showed mitochondrial internalization in DCX cells with a typical fusiform morphology.

## Discussion

Mitochondrial transplantation has been extensively studied in recent years, with efforts to apply it to treatments for a variety of mitochondrial and non-mitochondrial diseases^21,22^. Based on the pathogenesis of each disease, various invasive approaches have been used for transplantation, including intravascular injection, subcutaneous injection, and in situ injection^5,22,23^. Non-invasive mitochondrial transplantation, however, has rarely been studied due to certain limitations, including the inability to preserve mitochondrial activity after isolation, the lack of specificity in targeted delivery, and the problem of poor delivery efficiency. To the best of our knowledge, this is the first study to demonstrate the therapeutic feasibility of brain targeting via intranasal administration of allogeneic mitochondria in a neurotoxin-induced PD rat model. In addition, we utilized BrdU for mitochondrial labeling. BrdU is a useful labeling method that does not gradually lose signal strength, as does green fluorescent protein. BrdU can be used for long-term mitochondrial tracking and does not affect the morphology or activity of mitochondria^24,25^. In contrast to our previous study using a medial forebrain bundle (MFB) injection of mitochondria for PD treatment^7^, the present results show that when mitochondria were twice administered intranasally, restoration of mitochondrial complex I protein in substantia nigra neurons (~ 52%) was significantly lower than with local injection (~ 85%) using the same treatment frequencies and intervals. However, intriguingly, the improvement of animal behavior (except for the 40% lower index of cross-zoom frequency) and the increase in survival of nigra DA neurons were similar. This may be related to differences of mitochondrial uptake in specific regions of PD brains, due to different interventions. Indeed, neurons, astrocytes and glial cells of the cerebrum differed in mitochondrial internalization efficacy, which was also affected by different transplantation routes in the rescue model of stroke rats^25^. By tracking BrdU-labelled mitochondria, we found that mitochondria delivered intranasally could only be observed in the ST (the terminal of DA neurons) in contrast to mitochondria injected into the MFB, which were expressed in SN (somata of DA neurons)^7^. Chemogenetic stimulation of striatal projection neurons modulated responses to Parkinson’s disease therapy^26^ and this supports the notion that while the intranasal route for mitochondrial delivery had lower restoration efficacy for mitochondrial complex I protein of nigra DA neurons, it still had therapeutic effects similar to those of local injections.

In 2011, Danielyan et al. have demonstrated the therapeutic efficacy of intranasally delivered mesenchymal stem cells (MSCs) to the brain in a unilaterally 6-OHDA-lesioned rat model of Parkinson’s disease^10^. Although the delivery timing and frequency differed, delivered mitochondria and cells both resulted in higher performance in lesioned ST relative to lesioned SN and manifested a 60–80% improvement in motor function after > 70 days of treatment^10^. Interestingly, in a D-amphetamine-induced rotational behavior test to evaluate neuronal DA loss^27^, significant inhibition of rotational behavior occurred in the 11th week of continuous treatment with mitochondria versus in 136 days of end treatment with MSCs^10^. Mitochondrial treatment restored DA neuron viability sooner than MSCs treatment, though the restored level of TH in lesioned ST and SN was similar to stem cell treatment showed in Danielyan et al.^10^. In contrast to MSCs, the absence of delivered mitochondria in the SN makes it difficult to explain this phenomenon based on our limited data. We suggest that functional support of nigral DA neuron nerve terminals is more important in ST than in SN in PD therapy, because consistently higher expression level of delivered MSCs were observed in lesioned ST than in lesioned SN more than 6 months after treatment ended^10^. The latest article published in the period of our article being reviewed supports indirectly our finding that nasal administration of mitochondria reverses chemotherapy-induced cognitive deficits^28^. ST and its cortical connections is well-known critical regulator for cognitive symptoms^29^.

Compared to injected MFB mitochondria, which need to be modified for uptake into nigra DA neurons^11^, nose-to-brain delivery affords easier access to the brain since mitochondria do not need to be modified to penetrate the AOB and to enter the ST via the RMS pathway. The RMS pathway provides a “conduit” for intranasal mitochondrial delivery, guiding internalized mitochondria into the ST for restoration of dysfunctional DA neurons in PD. Moreover, naked mitochondria can also enter neurons by perfusion ipsilateral to the lesioned side and can be delivered to DA neurons of the ST on the contralateral, non-lesioned side via axons of interhemispheric commissures, such as the AC and CC^30^. Although contralateral delivery efficiency of naked mitochondria was not as high as that of peptide-modified mitochondria, therapeutic effects were not affected in the unilateral lesioned rat PD model used here. Further studies are required to determine whether better contralateral mitochondrial delivery efficiency would enhance therapeutic effects in bilateral SN lesions of PD model rats. It would be beneficial to increase delivery efficiency of mitochondria from nose-to-brain and to simplify the process of transplantation. In addition, studies have shown that proteins and nanoparticles can be delivered intranasally to brain parenchyma, the third and fourth ventricles, midbrain, and hippocampus, via the same pathway (the RMS pathway) in transgenic mouse models of Alzheimer’s disease^31^. However, in contrast to these techniques, which could also utilize intracellular transport of olfactory axons and extracellular transport processes along the olfactory nerve and the trigeminal nerve, mitochondria could only be delivered via the extracellular pathway to enter the AOB (since no significant presence of mitochondria was observed in olfactory neurons and the olfactory-trigeminal pathway, even after long-term treatments) and then reached the basal ganglia of the forebrain and lateral ventricles through the RMS pathway. This may be caused by variation in transport of mitochondria internalized in different types of neurons^32^. Otherwise, the diversity of intracellular signal transduction pathways in response to extracellular guide signals leads to variable cellular uptake of mitochondria^33^.

Moreover, we found that expression of plasma inflammatory cytokines was affected by different interventional approaches. Contrary to more serious contraindications resulting from local injections of vehicle relative to PD non-treatment controls^7^, high plasma inflammatory cytokine levels of PD rats were significantly reduced by intranasal vehicle perfusion, implying that this intervention will not exacerbate the disease during treatment^34^. Saline nasal irrigation is clinically proven for postoperative inflammation and to minimize antibiotic resistance^35^. One explanation for its utility is that the nasal inflammatory mediator, leukotriene C4, is substantially less elevated 2–6 h after treatment^35^. Clinical studies have shown that intranasal delivery of a placebo (saline) or an antioxidant enzyme (glutathione) alone over a three-month period provided symptomatic improvements in PD patients^36^ and anti-inflammatory benefits for PD^22^. Moreover, our results are consistent with our previous finding that modifying mitochondria with Pep-1 reduces their induction of plasma pro-inflammatory cytokines^7^. Although this outcome did not significantly affect therapeutic efficacy after just three months of treatment, further investigation is necessary to study its long-term post-operative effects. Moreover, while mitochondria delivered via brain injection induced higher levels of proinflammatory cytokines^7^, intranasal mitochondrial delivery only induced expression of interleukin (IL)-1α, IL-1β, IL-10, and IL-17A, further demonstrating the immunological safety of intranasal drug delivery.

## Conclusions

Various nose-to-brain delivery routes for nanomedicines, oligonucleotides, enzymes, and stem cells have been applied in PD treatments. This study demonstrates preliminarily that mitochondrial delivery from nose-to-brain is a feasible approach, and is safer than brain injection. While functional restoration of mitochondria to DA neurons using this approach is not as effective as by direct injection, neuronal survival and behavioral improvement are similar. Further, by this method, mitochondria can be delivered to the brain without modification. Unfortunately, mitochondria were only observed in the terminals of DA neurons in the ST and were not found in somata of DA neurons in the SN. Our previous studies have shown that injected mitochondria improve mitochondrial homeostasis and turnover by restoring dynamic mitochondrial protein loss in DA neurons, including increasing mitochondrial fusion-fission proteins and autophagy of damaged mitochondria in the SN^7^. Selectivity of mitochondrial fusion is particularly beneficial under conditions of increased mitochondrial damage, because it facilitates fusion frequency without compromising removal of damaged organelles by mitophagy, resulting in improved mitochondrial quality^37^. Whether this regulation is affected by different mitochondrial transplantation routes is under investigation. If retrograde transport of mitochondria from the minus-end in striatal terminal axons toward the cell body in the SN can be increased, therapeutic efficacy may be improved. Furthermore, therapeutic benefit of transfer of mtDNA-laden extracellular vesicles (EVs) have been reported not only in aggressive breast cancer^38^ but also in PD^39^. Thus, whether the presence of exogenous mtDNA in EVs from neurons rescused with mitochoindrial transplanation is worthy to study further to clarify the diversified roles of mitochondrial regulation on the PD pathogenesis.

## Methods

### Mitochondrial labelling and isolation

Allogeneic mitochondria were isolated from rat livers. For tracking of mitochondria after delivery, mitochondrial DNA (mtDNA) in hepatocytes was labeled with 5-Bromodeoxyuridine (BrdU) (Sigma-Aldrich, Burlington, MA, USA) prior to isolation. Rats were sacrificed 36 h after a single intraperitoneal (ip) injection of BrdU (50 mg/kg). Livers were homogenized in ice cold buffer (210 mM mannitol, 70 mM sucrose, 5 mM Tris–HCl and 1 mM EDTA, pH 7.4) using a Precellys homogenizer (Bertin Technologies, MD, USA). Through a series of filtration and centrifugation steps, as described previously^22^, isolated mitochondria were re-suspended in ice-cold MiR05 respiration buffer (0.5 mM EGTA, 3 mM MgCl_2_, 60 mM lactobionic acid, 20 mM taurine, 10 mM KH_2_PO_4_, 20 mM HEPES, 110 mM D-sucrose, 0.1% w/v fatty acid-free BSA) and prepared for use. BrdU immunofluorescence was used to track internalized mitochondria in sample tissue.

### Mitochondria conjugated with Pep-1

Detailed procedures for Pep-1 conjugation have been described previously^24^. 200 μg allogeneic mitochondria suspended in respiration buffer were conjugated with 0.11 mg Pep-1 diluted with sterile water (Anaspec, San Jose, CA, USA). Incubation for 10 min at room temperature was performed to ensure complex assembly.

### Animal study and nasal mitochondria administration

Adult female Sprague–Dawley rats (8 weeks old, weighing 250–300 × g) were purchased from BioLASCO (Taipei, Taiwan) and were maintained under standard laboratory conditions with free access to food and tap water in the Laboratory Animal Center, Changhua Christian Hospital, Changhua, Taiwan. All experimental procedures involving animals were performed in accordance with guidelines of the Association for Assessment and Accreditation of Laboratory Animal Care (AAALAC) and approval by the Animal Experiments and Ethics Committee of Changhua Christian Hospital (approval number CCH-AE-105–018). All animal and related experiments were carried out in compliance with the ARRIVE guidelines and followed its instructions of ARRIVE guidelines checklist. A detailed procedure for creating a neurotoxic rat model of Parkinson's disease using a unilateral injection of 6-OHDA into the medial forebrain bundle (MFB) has been described previously^7^. Successful induction of PD rats after three weeks was validated by a rotational behavior test, described below. PD rats were assigned randomly to five groups of six rats: 1) control group receiving no treatment (WT), 2) intranasal infusion of vehicle only (Sham), 3) mitochondria alone (Mito), 4) Pep-1-labelled mitochondria (P-Mito) and 5) Pep-1 alone (Pep-1). Rats received a unilateral, intranasal drop infusion (ipsilateral to the lesioned side) of 200 μg Mito or P-Mito in 50 µL MiR05 respiration buffer using a micropipette (QSP, 200 µL flter tips, Thermo Fisher Scientifc, Waltham, MA, USA) for three months treatment applied once a week. Rats were quickly and firmly picked up by the scruff of the neck behind the ears with thumb and index finger of hand and held in a supine position with the head elevated. The tip of the micropipette was placed 1–2 mm from the nostril entrance before introduction of nose drops into the nasal cavity while the rats were awake. Recording of intranasal administration of mitochondria is presented in Supplementary File 1. Experiments were designed to minimize the number of animals used and their suffering.

### Rotational behavior test

Motor imbalance of 6-OHDA-lesioned animals was assayed by methamphetamine (3 mg/kg, *i.p.*)-induced rotation. Rotational asymmetry was assessed using an automated rotometer system (AccuScan Instruments, Columbus, OH, USA) based on the design of Ungerstedt & Arbuthnott^40^ to record the counterclockwise rotation with full-body turns. Rats that exhibited more than 360 rotations within 60 min were randomly assigned for experimental use. During the period of the experiment, the rotating test was reperformed for 3 or 4 weeks post-treatment.

### Open field test

To assess general motor behavior, an open field test was employed 3 months after mitochondrial transplantation. Animals were placed in a 50 cm × 50 cm white plexiglass box and allowed an adaptation period of 30 min prior to being analyzed. Activity was recorded for two consecutive sessions, each lasting 15 min, using a ceiling-mounted video camera. Ethovision software (Noldus, Leesburg, VA, USA) (https://www.noldus.com/ethovision-xt) was used to measure distance, velocity, total number of zone boundaries crossed, and duration of movement (seconds). Locomotion frequencies were assessed by dividing the floor of the box into four quadrats and by counting the number of quadrats entered. Entry was counted when a rat entered a new quadrat with all four paws. The apparatus was washed with 5% ethanol between tests to eliminate possible bias due to odors left by previous rats.

### Histological and immunohistochemical staining

Rats that survived 3 months after transplantation were sacrificed with an overdose of chloral hydrate (800 mg/kg *i.p.*) and fixed by intracardiac perfusion with 300 mL of saline followed by 300 mL of 4% paraformaldehyde (Sigma-Aldrich). Brains were removed, post-fixed in 4% paraformaldehyde for 4 h, and cryoprotected in 30% w/v sucrose in PBS for 20 h. Brains were then frozen and embedded in OCT medium (Tissue-Tek, Sakura Finetek, USA) and sectioned at 5–10-μm.

For Nissl staining, sections mounted on glass slides were dried overnight. Slides were immersed in 0.025% cresyl violet (Sigma-Aldrich) in 90 mM acetic acid (Merck, Darmstadt, Germany) and 10 mM sodium acetate (Sigma-Aldrich) for 3 h, followed by dehydration in ascending ethanol and xylene series. Slides were then coverslipped with Histochoice mounting media (AMRESCO).

For immunohistochemical staining (IHC), fixed sections were subjected to heat-induced epitope retrieval in 10 mM citrate buffer pH 6.0 for 25 min at 100 °C. Sections stained with BrdU were additionally treated with 2 N HCl for 30 min at 37 °C. After washing and blocking non-specific sites with blocking buffer (5% BSA and 0.5% Tween-20 in PBS, pH7.4) for 30 min at room temperature, sections were incubated at 4 °C overnight with a primary antibody, anti-Tyrosine hydroxylase (TH) (1:200 dilution, Novus Biologicals, Littleton, CO, USA), anti-8-hydroxydeoxyguanosine (8-OHdG) (1:100 dilution, Japan Institute for the Control of Aging NIKKEN SEIL. Co., Ltd., Shizuoka, Japan), anti-BrdU (1:200 dilution, Abcam, Cambridge, MA, USA), or anti-Doublecortin (DCX) (Abcam) containing 1% BSA and 0.1% NaN_3_ (Sigma-Aldrich). Samples were then washed to remove excess first antibody and incubated for 30 min at room temperature with HRP-conjugated secondary antibody (1:200 dilution, Millipore) at a 1:200 dilution in PBS. After being washed three times in PBS to remove excess secondary antibody, chromogenic detection of immunoreactivity was performed using a DAKO 3,3'-diaminobenzidine (DAB) kit (DakoCytomation, Carpinteria, CA, USA) according to the manufacturer’s protocol. A counterstain, Mayer's hematoxylin (Sigma-Aldrich), was then added for 1 min. Color development was terminated by washing, and slides were visualized using light microscopy and image analyzed with Image J Software (NIH, Bethesda, MD, USA). For fluorescent detection in IHC, fluorophore-conjugated secondary antibodies (1:500 dilution in 0.5% BSA/PBS, Jackson ImmunoResearch, West Grove, PA, USA,) were used and nuclei were visualized with 4′,6-diamidino-2-phenylindole (DAPI) (Abcam) counterstaining. Fluorescent signals were detected and Z-stacks were acquired and analyzed with a confocal microscope (Olympus Fluoview FV1200, Olympus, Tokyo, Japan).

### Western blot analysis

A 30-μg aliquot of whole-cell lysate was fractionated on a 12% SDS–polyacrylamide gel (Bio-Rad Laboratories, Richmond, CA, USA) and blotted onto a polyvinylidene difluoride membrane (Amersham Biosciences, Buckinghamshire, UK). Nonspecific binding was blocked with 5% skim milk in Tris-buffered saline with 0.1% Tween 20 buffer (Sigma-Aldrich), and the membrane was incubated with a cocktail of monoclonal antibodies (1:800 dilution) purchased from Mitosciences (Eugene, OR, USA) for evaluation of oxidative phosphorylation complexes, including complex I (CI)-20 kDa subunit (NDUFB8), complex II (CII)-30 kDa subunit (SDHB), complex III-core 2 subunit (CIII)-48 kDa, complex IV-subunit I (CIV-I)-40 kDa, and complex V-subunit alpha (CV)-55 kDa. After incubation with a horseradish peroxidase-conjugated secondary antibody (1:50,000 dilution; Jackson ImmunoResearch), protein intensity was determined using an enhanced chemiluminescence reagent (Immobilon Western, Millipore). Bands were digitally scanned, and intensities were quantified using Gel-PRO Analyzer 3.0 software (Media Cybernetics, Rockville, MD, USA). Immunoreactivity of rabbit anti-glyceraldehyde-3-phosphate dehydrogenase antibodies (GADPH) (1:1000 dilution, Santa Cruz Biotechnology) was analyzed simultaneously as an internal control.

### Multiplex cytokine assay

Rat blood plasma was collected after 3 months of treatment and after addition of 1:100 diluted proteinase inhibitor cocktail (Millipore #539,134), samples were frozen at − 80 °C until testing. Multiplex cytokines in rat plasma were measured in duplicate using a Luminex platform (MAGPIX, Millipore, St. Charles, MO, USA) with the customized panel, MILLIPLEX MAP rat Cytokine/Chemokine Magnetic Bead (MILLIPLEX MAP kits, EMD Millipore, Billerica, MA, USA) and analyzed with MILLIPLEX analyst software (ViageneTech, Carlisle, MA, USA) according to the manufacturer's instructions. Plasma levels of granulocyte colony-stimulating factor (G-CSF), interleukin (IL)-1alpha (IL-1α), IL-4, IL-1beta (IL-β), IL10, IL-12(p70), interferon gamma (IFNγ) and IL17A were selected on the basis of significant upregulation resulting from mitochondrial transplantation^7^.

### Statistical analysis

All analyses were performed in triplicate or quadruplicate in each group of experiments. Biochemical data are presented as means ± standard deviations, except results of the animal behavior test, presented as means ± standard errors of the means. There were six animals per group. The two mitochondrial treatments were evaluated using paired Student’s t-tests and differences with *p* < 0.05 were considered statistically significant. All statistical analyses and graphics were made with GraphPad Prism 5.0 software (GraphPad Software Inc., San Diego, CA, USA).

## Supplementary Information

Supplementary Video 1.

Supplementary Information 1.

## Abbreviations

PD: Parkinson’s disease
Mito: Mitochondria
P-Mito: Pep-1-labelled mitochondria
mtDNA: Mitochondrial DNA
SN: Substantia nigra
ST: Striatum
CC: Corpus callosum
AC: Anterior commissure
AOB: Accessory olfactory bulb
OB: Olfactory bulb
RMS: Rostral migratory stream
GL: Glomerular layer
