# Supplementary material for: Intranasal delivery of mitochondria for treatment of Parkinson’s Disease model rats lesioned with 6-hydroxydopamine
Source: Sci Rep. 2021 May 19;11:10597. doi: 10.1038/s41598-021-90094-w (PMC8136477; doi:10.1038/s41598-021-90094-w)

Title: Intranasal delivery of mitochondria for treatment of Parkinson’s Disease model rats lesioned with 6-hydroxydopamine

Authors: Jui-Chih Chang, Yi-Chun Chao, Huei-Shin Chang, Yu-Ling Wu, Hui-Ju Chang, Yong-Shiou Lin, Wen-Ling Cheng, Ta-Tsung Lin, Chin-San Liu

Supplements

Fig. S1. Whole uncropped images of the original western blots with three independent samples of each group. The western blot analysis shown in Fig. 4 (Red color indicated the different arrangement order of group from submitted Fig. 4A) was calculated and quantified based on this ensemble of results. OXPHOS proteins in ETC complexes were analyzed by western blotting using a commercially available anti-total OXPHOS primary antibody cocktail. NDUFB8 (Complex I), NADH dehydrogenase [ubiquinone] 1 beta subcomplex subunit 8; SDHB (Complex II), Succinate dehydrogenase [ubiquinone] iron-sulfur subunit; UQCRC2 (Complex III), Cytochrome b-c1 complex subunit 2; MTCO1 (Complex IV), mitochondrially encoded cytochrome c oxidase I; and ATP5A (complex V), ATP synthase, H + transporting, mitochondrial F1 complex, alpha 1. *Abbr.* MW, molecular weight; KDa, kilodalton


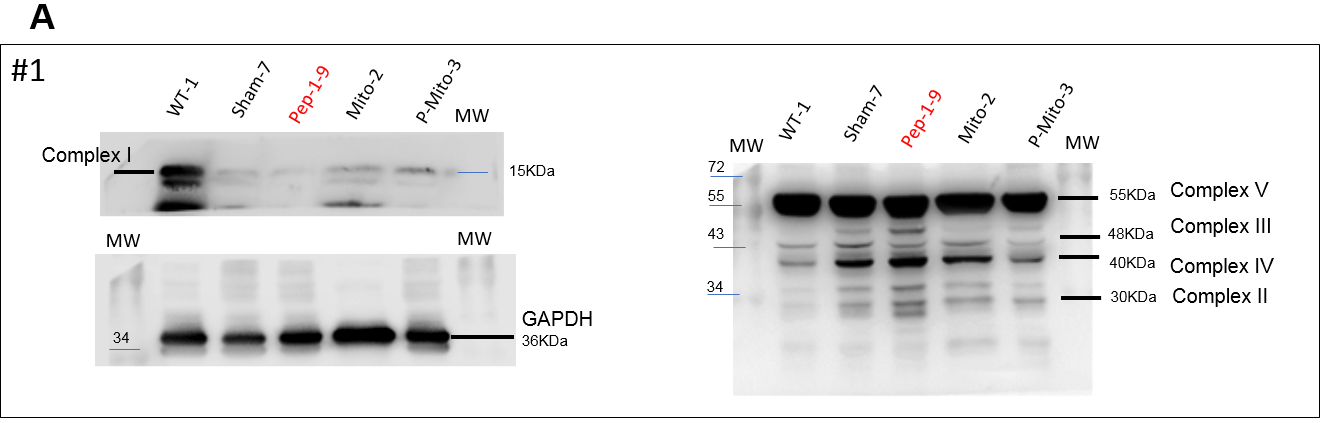


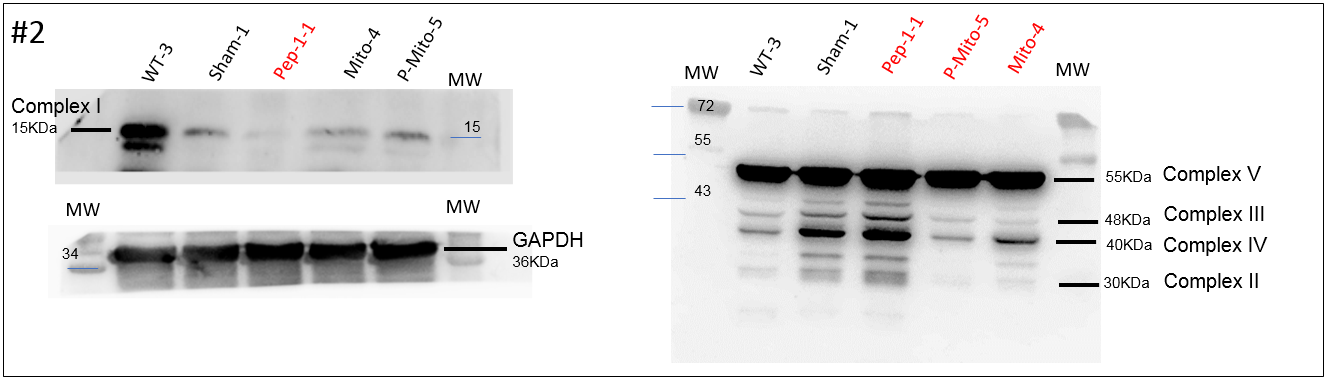


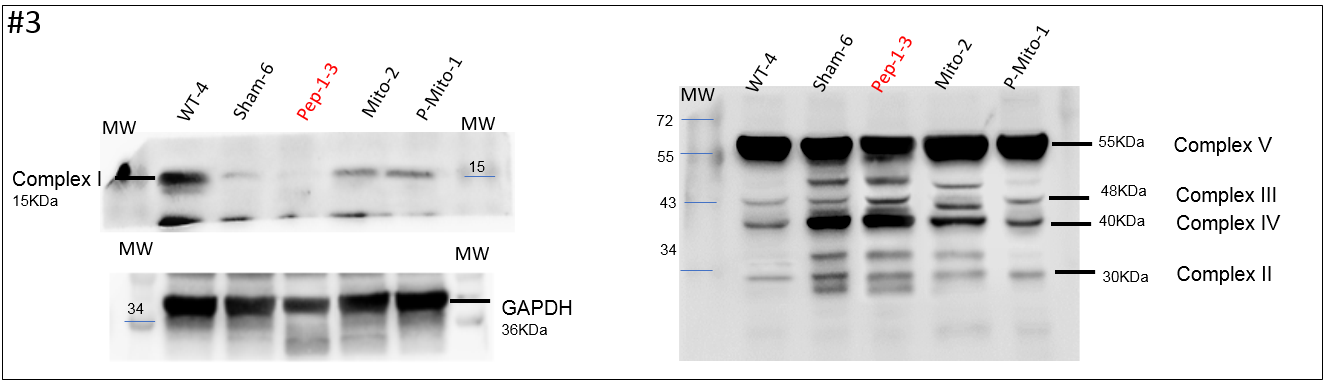

Supplement: Supplementary file 2 — Supplementary Information 1. [file 41598_2021_90094_MOESM2_ESM.docx]
